# The outcome of *Cryptococcus neoformans *intracellular pathogenesis in human monocytes

**DOI:** 10.1186/1471-2180-9-51

**Published:** 2009-03-05

**Authors:** Mauricio Alvarez, Tamika Burn, Yong Luo, Liise-anne Pirofski, Arturo Casadevall

**Affiliations:** 1 Department of Microbiology and Immunology and Medicine, Albert Einstein College of Medicine, 1300 Morris Park Ave., Bronx, NY 10461, USA

## Abstract

**Background:**

*Cryptococcus neoformans *is an encapsulated yeast that is a facultative intracellular pathogen. The interaction between macrophages and *C. neoformans *is critical for extrapulmonary dissemination of this pathogenic yeast. *C. neoformans *can either lyse macrophages or escape from within them through a process known as phagosomal extrusion. However, most studies of intracellular pathogenesis have been made with mouse cells and their relevance to human infection is uncertain. In this study we extended studies of *C. neoformans*-macrophage cellular interaction/s to human peripheral blood monocytes.

**Results:**

This study demonstrated that *C. neoformans *can shed polysaccharide within human monocytes, spread from cell to cell, and be extruded from them. Furthermore, human monocytes responded to ingestion of *C. neoformans *with cell cycle progression from G1 to S.

**Conclusion:**

Similarities between mouse and human cells support the suitability of mouse cells for the study of intracellular pathogenesis mechanisms. Given that these hosts diverged over 70 million years ago, the similar pathogenic strategies for *C. neoformans *in murine and human cells supports the hypothesis that the mechanism that underlies the mammalian intracellular pathogenesis of *C. neoformans *originated from interactions with a third host, possibly soil amoeboid predators, before the mammalian radiation.

## Background

*Cryptococcus neoformans *is an encapsulated yeast that is a facultative intracellular pathogen and a frequent cause of human disease in immunocompromised patients [1,2]. Macrophages are essential for effective host defense against *C. neoformans *in humans [3,4]. However, murine macrophages have been shown to be permissive for intracellular replication of *C. neoformans*, which can subsequently be extruded from or lyse the macrophages [2,5-8]. In this regard, *C. neoformans *has a unique intracellular pathogenic strategy that involves cytoplasmic accumulation of polysaccharide-containing vesicles and intracellular replication leading to the formation of large phagosomes where multiple Cryptococcal cells are present [5]. Our group and others have recently reported that after *C. neoformans *ingestion by macrophages, the yeast replicates and is subsequently extruded, in a process whereby both the yeast and macrophages survive [8,9]. Moreover, it was also recently discovered that *C. neoformans *can spread from an infected to an uninfected murine macrophage cell [9,10]. Here we further extend our extrusion studies to human peripheral blood monocytes (HPBMs) and report that as in murine macrophages, the interaction between human monocytes and *C. neoformans *leads to ingestion, intracellular replication, and polysaccharide shedding of *C. neoformans*, followed by cell to cell spread and extrusion of *C. neoformans*. The occurrence of phagosomal 'extrusion' in human peripheral blood monocytes suggests a central role for this phenomenon in the propagation and dissemination of this fungal pathogen.

*C. neoformans *has a novel intracellular strategy that, to date has no precedent in other well-characterized intracellular pathogens. Since *C. neoformans *is an environmental microbe that does not require a mammalian host for replication or survival, its sophisticated intracellular pathogenic lifestyle suggests that the mechanisms that govern its virulence are unique and distinct from microbes that require such a host, most likely stemming from selection in the environment [11]. In this regard, the discovery of similar interactions between *C. neoformans *and *Acanthamoebae castellanii *and *Dictiostelyium discoidum *and murine macrophages [12,13] have led to the hypothesis that the ability of *C. neoformans *to survive in mammalian cells evolved accidentally, perhaps from interactions with soil predators [11,14,15]. A corollary of this hypothesis is that the interactions of *C. neoformans *with cells from any mammalian species should be similar. In this study, we explore this corollary by studying *C. neoformans *interactions with human peripheral blood monocytes and show that these are similar to those described for murine macrophages.

## Results

### *C. neoformans *replicates and sheds polysaccharide in human peripheral blood monocytes

**Figure 1:**
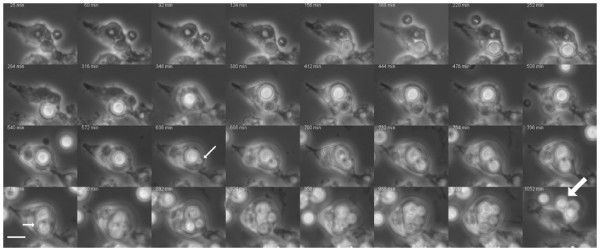
**Intracellular replication leads to extrusion of *C. neoformans *phagosome**. HPBMs were incubated with *C. neoformans *strain H99. Following incubation, *C. neoformans *budding occurred every 2–3 hours as evidenced by the small arrows. This was followed by extrusion of the *C. neoformans *phagosomes as evidenced by the large arrow. Images were collected at 10×.

**Figure 2:**
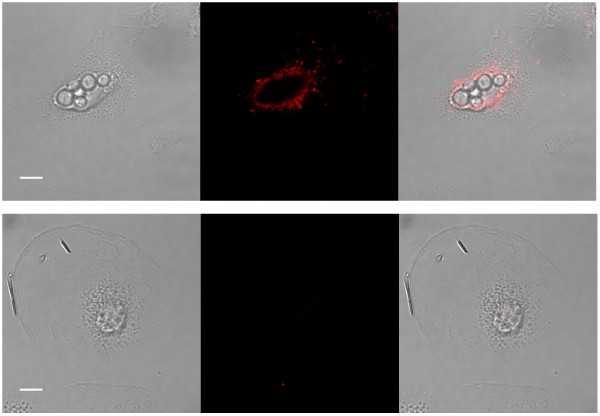
**Intracellular polysaccharide shedding by *C. neoformans *cells**. Polysaccharide shedding capacity of *C. neoformans *strain H99 was tested in HPBMs. Top panel: Intracellular shedding of cryptococcal polysaccharide from *C. neoformans *cells into HPBMs after 18 h incubation. Bottom panel: HPBMs lacking intracellular cryptococcal cells showed no fluorescence. Bar = 10 μM

*C. neoformans *replicated in HPBM cells at similar rates to extracellular *C. neoformans*, that is, every 2 to 3 h (Figure 1, See additional file 1: Movie 1). To investigate whether polysaccharide-filled vesicles formed following HPBM incubation with *C. neoformans*, HPBMs with and without ingested *C. neoformans *cells were permeabilized and incubated with conjugated Alexa 546-18B7, which binds GXM. The cells were then examined in a confocal microscope for the presence of cytoplasmic vesicles containing polysaccharide. As in previous studies, vesicles positive for polysaccharide were identified starting at 18 h post infection (Figure 2A). A group of control-uninfected cells gave no positive signal even when overexposed (Figure 2B).

### Cell-to-cell spread and extrusion of *C. neoformans *by HPBMs

**Figure 3:**
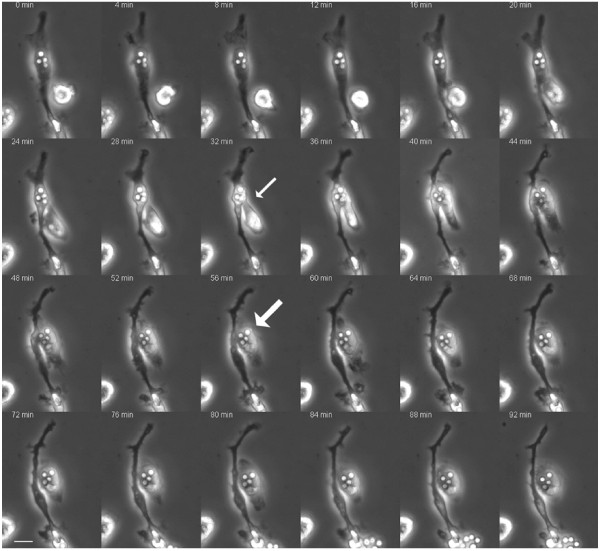
**Cell-to-cell spread of *C. neoformans *leads to infection of previously uninfected cell**. Following phagocytosis, human peripheral blood monocytes closely apposed to each other underwent fusion leading to cell to cell spread of *C. neoformans*. The small arrow points to the uninfected monocyte approaching the infected monocyte to sequester the yeast cells while the large arrow indicates the *C. neoformans *cells that have been fully transferred to the previously uninfected human monocyte. Bar = 10 μM

To study the occurrence of cell-to-cell spread and extrusion of *C. neoformans*, we incubated HPBMs with the yeast cells. Following ingestion and subsequent imaging, we witnessed that *C. neoformans *also spread from host human monocyte to another uninfected one (Figure 3) (See additional file 2: Movie 2), confirming similar observations made in other studies [7-10]. We also observed that *C. neoformans *was extruded from HPBMs in a similar fashion, as previously described for murine cells, leading to the survival of the yeast cells and the monocyte, as evidenced by continual budding and pseudopodial movements, respectively (Figure 1) (See additional file 1: Movie 1). Overall, out of 27 infected cells, 2 cell to cell spread events and 6 extrusion events were observed.

### Cell cycle distribution of monocytes is altered after Fc- and complement-mediated phagocytosis

**Figure 4:**
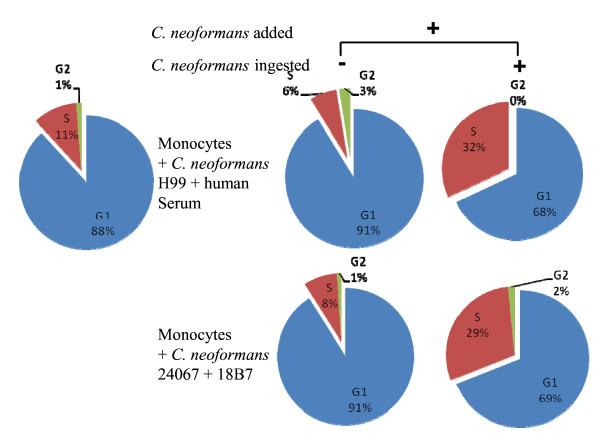
**Fc- and complement-receptor activation stimulates cell cycle progression of human peripheral blood monocytes from G1 to S**. Phagocytosis of *C. neoformans *strain 20467 mediated by 18B7 and *C. neoformans *strain H99 mediated by human serum was followed by an increase in S phase cell distribution of human monocytes. Percentage of G1, S and G2 cells are indicated in the control group (*C. neoformans *added – and *C. neoformans *ingested -) and the phagocytosis assay group (*C. neoformans *added +) which was further separated into the non-phagocytic (*C. neoformans *added + and *C. neoformans *ingested -) and the phagocytic (*C. neoformans *added + and *C. neoformans *ingested +) groups. Comparison of G1, S and G2 percentages between non-phagocytic and phagocytic groups revealed statistically significant differences (p < 0.001).

Previous studies with mouse cells reported an increase in S phase cells after complement and Fc-mediated phagocytosis of polystyrene beads, live or heat-killed *C. neoformans *[16]. Thus, we investigated whether the same phenomenon could be observed in primary human monocytes. We found that the majority of monocytes were in G1 phase in our culture conditions (88%) (Figure 4). Just as in cultured J774.16 cells, monocytes phagocytosed *C. neoformans *strain 24067 opsonized with mAb 18B7 and H99 opsonized with human serum. Both Fc- and complement-mediated phagocytosis resulted in cell populations that had a significant shift in cell cycle such that the monocytes with ingested *C. neoformans *had a much greater percentage of cells shifted into S phase relative to the population that did not phagocytose *C. neoformans *or relative to control cells that were unexposed to *C. neoformans *(Figure 4). Interestingly, in both phagocytosis assay groups, there was approximately a 20% decrease in the percentage of G1, which was greater compared to our previous report on J774.16 cells in which a 10% decrease in the percentage of G1 was observed (Figure 4) [16].

## Discussion

Blood-derived monocytes have been extensively used to study the interaction of *C. neoformans *with human phagocytic cells [17-19]). However, the mechanisms of cryptococcal intracellular pathogenesis have been studied largely with murine cells [2,6-10,20,6]. In this study, we investigated whether the events that characterized *C. neoformans*-murine macrophage interactions also occurred in human cells, with particular emphasis on fungal cell exocytosis, host cell cycle response, and intracellular polysaccharide shedding. This question is important because, in addition to validating observations made with murine cells in human cells, it can support or refute proposals for the emergence of cryptococcal virulence in mammalian hosts. If *C. neoformans *virulence for mammals did emerge accidentally from interactions with phagocytic predators in the environment one could posit that its interaction with macrophages from different mammalian species would be similar. To date *C. neoformans *interactions with mammalian macrophages have been limited to three species: mice, rats, and humans. The comparison of *C. neoformans *interactions with murine and rat macrophages was not revealing in this regard because the latter were a non-permissive host for cryptococcal replication [3]. Furthermore, there are mouse strain differences in murine macrophage permissiveness to cryptococcal replication that correlate with strain susceptibility to cryptococcosis [21]. Human monocytes are known to be permissive to *C. neoformans *intracellular replication [22,23], but the outcome of this interaction has not been explored. The major finding of this study is that the interaction of *C. neoformans *with human monocytes parallels that described with murine macrophages with regards to replication time, fungal cell exocytosis, phagocytosis-triggered cell cycle progression and intracellular polysaccharide shedding. These observations support the hypothesis that the mechanism of intracellular aggression for *C. neoformans *is conserved between amoebae to mice to humans

Cell replication is affected by external stimuli, such as growth factors, cell-cell contact, and cell adhesion to the extracellular matrix [16]. The fact that there was a 2-fold greater increase in human monocytes going to S phase (20% decrease of the percentage of G1) than in murine tissue macrophages (10% decrease of the percentage of G1) suggests that monocytes have a higher replication potential, which is consistent with the fact that they are less differentiated blood macrophage precursors. The consequence of phagocytic cell replication for the outcome of infection is not known. A greater ability to replicate could increase the number of effector cells as an outcome that could be advantageous to the host. On the other hand, the observation that *C. neoformans *growth in monocytes can exceed growth in media in the presence of opsonizing antibody [22], raising the possibility that host cell replication could be a disadvantage, since this would also increase the number of host cells available for intracellular infection and possibly, potentiate dissemination. Indeed, extrapulmonary dissemination in mice has been associated with macrophage ingestion in mice [24]. Induced stimulation of monocyte cell cycle progression following phagocytosis of *C. neoformans *could influence the outcome of infection by generating additional uninfected effector cells at the site of infection, as previously proposed by Luo, et. al., [16].

In this study we observed phagosomal extrusion in both forms, i.e. where single *C. neoformans *cells were extruded and complete extrusion, where all *C. neoformans *cells were extruded concomitantly. Single cell exocytosis from human monocytes was observed by Ma et al [8] but phagosome extrusion has not been previously reported in human cells. The significance of the observation that cell-to-cell spread and extrusion of *C. neoformans *occurred in human monocytes is that these events might contribute to disease pathogenesis, especially in immuno-compromised individuals where the proper cell-mediated immune response is lacking. Even though spreading of macrophages-ingested *C. neoformans *to other cell types has not been demonstrated it is nevertheless possible that it could take place and thereby contribute to the overall pathogenicity of *C. neoformans*. Further, human monocytes might function as 'Trojan horses' and deliver *C. neoformans *to the central nervous system, as described for HIV [25].

Our study, like all *in vitro *studies, has several limitations. First, human monocytes are macrophage precursors and consequently not fully differentiated. This could account for the significantly higher proportion of cells that underwent cell cycle progression upon *C. neoformans *phagocytosis relative to what was observed previously for murine macrophages. Second, isolated cells in tissue culture conditions could behave differently than in the body and consequently, one must be cautious in extrapolating these findings to *in vivo *situations. In this regard, the interaction of human monocytes with Cryptococcus is known to be highly dependent on the conditions of the experiment [22]. Third, we opsonized *C. neoformans *with a murine IgG1, an isotype that is known to engage human Fc receptors and promote phagocytosis. However, murine and human IgG could engage different types of receptors and it is conceivable that different results would be obtained with human IgG mAbs that are unfortunately not available.

Despite the limitations inherent in this system, we believe the similarities between *C. neoformans*-macrophage interactions for human and mouse cells is a significant result from the viewpoint of understanding the origin and range of cryptococcal virulence. This finding supports the continued use of mice and mouse cells for studies of certain *C. neoformans*-host interactions. Furthermore, the demonstration that the same phenomena that define cryptococcal pathogenesis in mouse cells also occur in human cells, provides strong support for central corollary of the 'ready-made virulence' hypothesis [15], namely, that the virulence of certain environmentally-acquired fungi is conserved across mammalian species. Given that humans and rodents diverged over 70 million years ago [26], the similarities in the intracellular pathogenesis of *C. neoformans *in mouse and human cells suggest two possibilities, which are not mutually exclusive. First, *C. neoformans *could be endowed with an ancient intracellular pathogenic mechanism that predated the mammalian radiation. Second, *C. neoformans *has a non-specific intracellular mechanism that allows it to survive and replicate in phylogenetically different phagocytes. These possibilities cannot be distinguished based on the available information. The fact that rat macrophages are not as permissive to *C. neoformans *replication as murine and human cells appears to be a function of more powerful antifungal mechanisms, which inhibit fungal growth [3]. Given that protozoa branched earlier than animals and fungi from the eukaryotic tree of life [27] and that fungi predate the emergence of animals in the evolutionary record, the similarities between the intracellular pathogenic strategy of *C. neoformans *for animals and protista are consistent with the view that cryptococcal virulence evolved to facilitate resistance to environmental predators to survive against said predators.

In summary, we establish that the interaction of *C. neoformans *with human monocytes is very similar to that described earlier for murine cells. The continuity in the phenomena observed for *C. neoformans *interactions with primate and murine cells highlights the importance of comprehensively studying the pathogenic strategy of *C. neoformans *in light of the innate immune defense.

## Conclusion

In summary, we establish that the interaction of *C. neoformans *with human monocytes is very similar to that described earlier for murine cells. The continuity in the phenomena observed for *C. neoformans *interactions with primate and murine cells highlights the importance of comprehensively studying the pathogenic strategy of *C. neoformans *in light of the innate immune defense.

## Methods

### Yeast Strains and Culture Conditions

*C. neoformans *var. *grubii *strain H99 was obtained from John Perfect (Durham, NC) and was cultured in Sabouraud dextrose broth (Difco) at 30°C with agitation (150–180 rpm).

### Murine macrophages

The macrophage-like murine cell line J774.16 derived from a reticulum sarcoma [28,29], was used for some of the experiments. Macrophages were collected by centrifugation, and re-suspended in feeding media consisting of Dulbecco's minimal essential medium (DMEM) (Life Technologies), 10% NCTC-109 medium (Gibco), 10% heat-inactivated (56°C for 30 min) FCS (Gemini Bio-products, Woodland, CA, USA), and 1% non-essential amino acids (Mediatech Cellgro, Washington, DC, USA). Cells were then plated on poly-lysine coverslip-bottom MatTek plates (Ashland, MA) at a density of 5 × 10^4 ^per well in feeding media and allowed to adhere overnight at 37°C and 10% CO_2 _prior to incubation with *C. neoformans *for an additional 1 hr and subsequent microscopic imaging.

### Collection of human peripheral blood monocytes and phagocytosis

Monocytes were isolated by Ficoll-Hypaque (GE Healthcare, Piscataway, NJ) density gradient centrifugation as described previously [30]. Briefly, diluted venous blood from one healthy donor was diluted with Hank's balanced salt solution (Mediatech, Herndon, Va) and was layered on top of Ficoll-Hypaque (GE Healthcare) at a 1:1 ratio and centrifuged at 2000 rpm/4°C for 15 minutes without brake. The monocyte layer was removed and red blood cells were lysed using lysing buffer (0.155 M NH_4_Cl pH 7.4). Cells were washed three times with Hank's balanced salt solution and suspended in RPMI (Mediatech) media supplemented with 10% fetal calf serum (Gemini Bioproducts, West Sacramento, Ca) and cells were then plated on poly-lysine coverslip-bottom MaTtek plates (Ashland, MA) at a density of 2 × 10^5 ^per well in feeding media and allowed to adhere at 37°C and 10% CO_2 _for 6 days prior to incubation with *C. neoformans*, using 18B7 (10 ug/ml) or 20% human serum, for 1 hr and subsequent microscopic imaging. This study was done with the approval of our institutional review board committee at the Albert Einstein College of Medicine and prior consent was obtained from blood donors.

### Time-lapse imaging

For live cell imaging, phagocytosis assays were done as described [9]. Briefly, 10^5 ^HPBM were plated on polylysine coated coverslip bottom MatTek plates and allowed to adhere for 6 days. The media was then removed and replaced with fresh media containing *C. neoformans *cells (*C. neoformans *to HPBM ratio of 10:1) along with monoclonal antibody (mAb) against the cryptococcal capsule (mAb 18B7, 50 μg/ml). *C. neoformans *were opsonized with either mAb 18B7 or 20% guinea pig serum as indicated above. HPBMs and *C. neoformans *were then incubated together for 30 min at 4°C to synchronize phagocytosis, followed by 60 min incubation at 37°C to allow for completion of phagocytosis. This was followed by two washes with fresh media (1 ml each), and replenishment with 2 ml feeding media. The plates were then taken for time-lapse imaging every 4 minutes using an Axiovert 200 M inverted microscope and photographed with an AxiocamMR camera controlled by the Axio Vision 4.4 software (Carl Zeiss Micro Imaging, NY). This microscope was housed in a Plexiglas box and the temperature was stabilized at 37°C with a forced air heater system. The plate lid was kept in place to prevent evaporation, and 5% CO_2 _was delivered to a chamber locally at the culture dish. Quantitative analysis of phagosomal extrusion and cell to cell spread was carried out by compiling all the movies and counting the number of macrophages with internalized *C. neoformans *and the number of phagosomal extrusion or cell to cell spread events from these macrophages, followed by the determination of the percentage of cells that had extruded the *C. neoformans *containing phagosomes or had transferred at least one cryptococcal cell to another cell nearby {(macrophages that extruded phagosomes ÷ macrophages with internalized *C. neoformans*) × 100}. Movie animations were created using ImageJ software [31]. To assess intracellular replication, live-cell time lapse imaging was initiated immediately after initial incubation of macrophages with *C. neoformans *and was measured up to two successive rounds of *C. neoformans *replication. Images were collected at 40×.

### Confocal imaging

Phagocytosis was carried out as indicated above, and after 18 h, human peripheral blood monocytes and *C. neoformans *were fixed with 4% paraformaldehyde for 10 min followed by a 5 min permeabilization with 1% Triton-X 100. Labeling of *C. neoformans*' capsular polysaccharide was achieved with 18B7 conjugated to Alexa-546, according to the manufacturer's instructions (Molecular Probes). Samples were then suspended in mounting medium (50% glycerol and 50 mM *N*-propyl gallate in PBS) and visualized using a Leica AOBS laser scanning confocal microscope. Z-series images were collected using a 63×/1.4 Oil objective. Minor processing adjustments were made using Adobe Photoshop CS2.

### Phagocytosis assay coupled with flow-cytometric analysis

Human peripheral blood monocytes were cultured in 6-well plates to a density of 1 × 10^5 ^to 2 × 10^6 ^cells per well. In Fc-mediated phagocytosis assays, antibody-opsonized *C. neoformans *strain 24067 was added at an effector to target ratio of 1:1. *C. neoformans *capsule-specific mAb, 18B7, was used as an opsonin at 10 μg/ml. In complement-mediated phagocytosis assays, FITC-labeled *C. neoformans *strain H99 was added at an effector to target ratio of 1:1 and 20% human serum was added to promote phagocytosis. Incubation was carried out in 10% CO_2 _at 37°C. After incubating for 1.5 h, any remaining extracellular yeast cells were removed with three washes of PBS.

The macrophage monolayer was gently scraped from the 6-well plates and suspended in 1 ml PBS for each well. Cells were fixed by the addition of 5 ml ice-cold 70% ethanol, and incubated on ice for 2 h. In preparation for FACS analysis, cells were centrifuged at 600 rpm for 10 min. DNA content was labeled by incubating the pellets in a 0.5 ml solution of propidium iodide (Molecular Probes, Eugene, OR) at 20 μg/ml in PBS, containing RNAse at a final concentration of 200 μg/ml. Samples were stained at room temperature for 30 min and analyzed by FACScan (Becton-Dickinson, Mountain View, CA). J774 cells incubated with particles were sorted into the non-phagocytic population and the phagocytic population according to absence or presence of intracellular FITC signal from 18B7 conjugated with Alexa 488 or *C. neoformans *strain H99 which was labeled with FITC. Data were analyzed by ModFit 3.0 software (Verity Software House, Topsham, ME) for cell cycle distribution. The distribution of cell cycle stages in each population was compared. The experiments were independently repeated three times.

## Authors' contributions

MA carried out the bulk of the work reported in this article. TB collected the Peripheral blood human monocytes, and YL carried out the FACS experiments. AC and LP envisaged the work in the manuscript and helped prepare the manuscript. All authors' read and approved the final manuscript.

## Authors' information

Mauricio Alvarez, Yong Luo and Tamika Burns are graduates of the Albert Einstein College of medicine. Arturo Casadevall is chairman of the microbiology & immunology department at the Albert Einstein College of Medicine. Liise-anne Pirofski is professor of medicine, microbiology and immunology and is chief of the Division of Infectious Diseases at Einstein.

## Supplementary Material

Additional file 1 **Replication of *C. neoformans *within human peripheral blood monocytes.** The data provided represents intracellular replication of *C. neoformans *in HPBM cells at rates similar to extracellular *C. neoformans *(every 2 to 3 h). Click here for file

Additional file 2 **Cell to cell spread of *C. neoformans *in human peripheral blood monocytes.** Cell to cell spread was witnessed following ingestion and subsequent imaging of infected HPBMs, we witnessed that *C. neoformans *also spread from host human monocyte to another uninfected one. Click here for file
